# Multifaceted effects on *even-skipped* transcriptional dynamics upon *Krüppel* dosage changes

**DOI:** 10.1242/dev.202132

**Published:** 2024-03-04

**Authors:** Shufan Lin, Bomyi Lim

**Affiliations:** ^1^ Department of Bioengineering, University of Pennsylvania, Philadelphia, PA 19104, USA; ^2^ Department of Chemical and Biomolecular Engineering, University of Pennsylvania, Philadelphia, PA 19104, USA

**Keywords:** Transcriptional regulation, Live imaging, *Drosophila*, *Even-skipped*, *Krüppel*, MS2

## Abstract

Although fluctuations in transcription factor (TF) dosage are often well tolerated, TF dosage modulation can change the target gene expression dynamics and result in significant non-lethal developmental phenotypes. Using MS2/MCP-mediated quantitative live imaging in early *Drosophila* embryos, we analyzed how changing levels of the gap gene *Krüppel* (*Kr*) affects transcriptional dynamics of the pair-rule gene *even-skipped* (*eve*). Halving the *Kr* dosage leads to a transient posterior expansion of the *eve* stripe 2 and an anterior shift of stripe 5. Surprisingly, the most significant changes are observed in *eve* stripes 3 and 4, the enhancers of which do not contain Kr-binding sites. In *Kr* heterozygous embryos, both stripes 3 and 4 display narrower widths, anteriorly shifted boundaries and reduced mRNA production levels. We show that *Kr* dosage indirectly affects stripe 3 and 4 dynamics by modulating other gap gene dynamics. We quantitatively correlate moderate body segment phenotypes of *Kr* heterozygotes with spatiotemporal changes in *eve* expression. Our results indicate that nonlinear relationships between TF dosage and phenotypes underlie direct TF-DNA and indirect TF-TF interactions.

## Teaser

**Summary:** Changes in transcriptional dynamics of *even-skipped* upon halving *Krüppel* dosage reveal how the combination of direct transcription factor (TF)-DNA and indirect TF-TF interactions shape proper patterning.

## INTRODUCTION

Body segmentation is a crucial process that specifies repeated units along the anterior-posterior (AP) axis during early embryogenesis. Although detailed processes differ among species, body segmentation is a conserved strategy for creating blueprints to guide the formation of structures and organs (Akam, 1987; Diaz-Cuadros et al., 2021; Saga and Takeda, 2001). In *Drosophila*, body segment formation is completed through a hierarchical gene regulatory network that encodes cascades of transcription factors (TFs) (Ingham, 1988; Jaeger, 2011; Schroeder et al., 2011). *bicoid* (*bcd*), *nanos* and products of other maternal effect genes are first localized to either the anterior or posterior end and polarize the embryo, establishing sequential gap gene expression domains in a concentration-dependent manner along the AP axis (Driever and Nüsslein-Volhard, 1988; Ephrussi and Johnston, 2004; Rivera-Pomar et al., 1995; Wang et al., 1994). Refined expression of gap genes gives rise to distinct stripes of pair-rule genes in nuclear cycle 14 (NC14) (Goto et al., 1989; Surkova et al., 2008). In later stages, pair-rule genes regulate segment-polarity genes and complete the body segmentation process (DiNardo and O'Farrell, 1987; French, 2001). The spatiotemporal dynamics of upstream TFs regulate the next cascade of TFs, establishing the highly reproducible body patterning process during early development. Transcriptional and translational regulation of TFs should be tightly controlled to proceed with the next layer of the gene regulatory network, whereas a certain level of resistance to variability or a compensatory mechanism is also in place to ensure normal development.

*Drosophila* often exhibits a high tolerance to TF dosage perturbations. Flies carrying three or four copies of *bcd* can buffer the high dosage through the interplay among downstream gap genes and develop into normal adults (Berleth et al., 1988; Liu et al., 2013). In addition, heterozygous mutants for most patterning genes are viable and fertile (Driever and Nüsslein-Volhard, 1988; Surkova et al., 2019; Yu and Small, 2008). On the other hand, such high tolerance does not necessarily imply the robustness of embryonic development. Some larvae that are heterozygous for *hunchback* (*hb*) or *Krüppel* (*Kr*) have partial body segment deletions (Lehmann and Nüsslein-Volhard, 1987; Wieschaus et al., 1984). Phenotypic differences due to moderate changes in TF dosage were also observed in other species. Although a heterozygous mutation of a TF *Sox9* does not cause embryonic lethality in humans and mice, its reduced dosage drives skeletal malformations at the lower jaw (Long et al., 2020; Naqvi et al., 2023). These results indicate that organisms show some degree of robustness to changes in TF dosages, preventing lethal outcomes. However, the expression of downstream genes can still be altered upon TF dosage modulations and lead to significant non-lethal phenotypes. Genome-wide human genetics studies have also identified many genetic variants that are associated with phenotypic traits, and such variants often modulate the level of TFs (Gupta et al., 2021; Maurano et al., 2012). These recent findings emphasize the need to better understand the molecular impact of TF dosage in gene regulation and subsequent phenotypes.

We use the highly dynamic interactions among gap genes and pair-rule genes to study the role of TF dosage in patterning and developmental robustness. Both gap and pair-rule genes undergo stochastic transcription at the beginning of NC14, followed by the formation and refinement of sharp expression domains in 50 min (Jaeger et al., 2004; Perry et al., 2012; Surkova et al., 2008). Although flies with reduced gap gene copies are viable, little is known about how the change in TF dosage affects the dynamics of pair-rule gene regulation and subsequent developmental robustness. Does a decrease in TF concentration drive ectopic target gene expression? Are these changes connected to the defects observed in some heterozygotes from previous studies? With a real-time, high spatiotemporal resolution platform, we can quantify subtle changes in transcriptional activity to elucidate the molecular mechanism underlying how fluctuations in TF dosage lead to phenotypic consequences.

In this study, we have used MS2/MCP- and PP7/PCP-based live imaging and genetic perturbations to examine the direct and indirect role of the gap gene *Kr* in the transcriptional regulation of a pair-rule gene *even-skipped* (*eve*). We quantified distinct changes of different *eve* stripes in response to the decrease in *Kr* dosage. The observed shift in *eve* stripe 2 and 5 boundaries is likely due to a weaker Kr binding to the respective enhancers. Surprisingly, the most affected domains are *eve* stripes 3 and 4, even though their enhancers lack Kr-binding sites (Castro-Mondragon et al., 2022; Li et al., 2008; Paris et al., 2013). Decreased Kr shifts stripes 3 and 4 boundaries anteriorly, narrows the expression domain and reduces mRNA production. We further demonstrate that these effects on stripes 3 and 4 are propagated by the changes of adjacent gap genes, *giant* (*gt*), *knirps* (*kni*) and *hb*, in *Kr* heterozygotes. By quantitatively analyzing how individual nuclei respond to fluctuations in Kr concentration, our results provide insights into the systematic interplay among multiple genes in determining body pattern formation. Moreover, our study presents a quantitative and mechanistic analysis on the impact of TF dosage modulation in regulating key developmental genes.

## RESULTS

### Decreased *Kr* level leads to higher variability in *eve* expression pattern

**Fig. 1.**
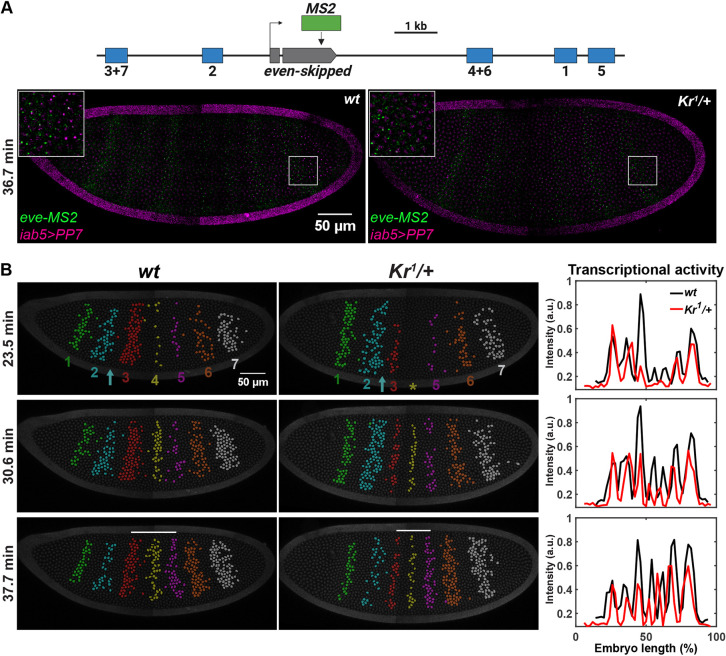
**Halved *Kr* dosage affects the overall transcriptional dynamics of *eve*.** (A) Top: schematic of the *eve-MS2* construct. Bottom: snapshots of a wild-type and *Kr* heterozygous embryo expressing *eve-MS2* (green) and *iab5>PP7* (magenta) in NC14. The insets are magnifications of the outlined regions. (B) Snapshots of a wild-type and *Kr* heterozygous embryo expressing *eve-MS2*. Transcriptionally active nuclei are false-colored. Nuclei are labeled with His2Av-eBFP2 (dark gray). Yellow asterisk indicates the position of *eve* stripe 4; cyan arrows indicate the region between stripes 2 and 3; white bars indicate the distance between stripes 3 and 5. The graphs show the transcriptional activity of *eve-MS2* across the AP axis in the embryos on the left.

**Fig. 2.**
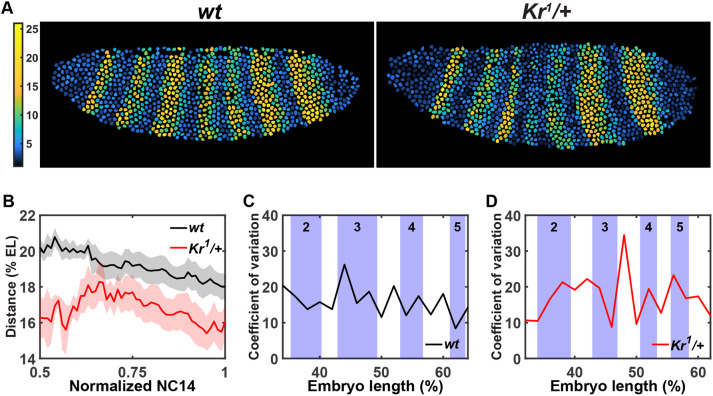
**Halved *Kr* dosage reduces mRNA output and increases variability in the *eve* stripe 2-5 region.** (A) Heatmaps showing cumulative mRNA production in individual nuclei in a wild-type (left) and a *Kr* heterozygous (right) embryo. (B) Average distance between *eve* stripes 3 and 5 over time. Normalized NC14 represents the time between the end of 13th mitosis (t=0) and the beginning of gastrulation (t=1). Data are mean±s.e.m. of wild-type (*n*=7) and *Kr* heterozygous (*n*=5) embryos. (C,D) Coefficient of variation along the AP axis across *eve* stripes 2-5 region in seven wild-type (C) and five *Kr* heterozygous (D) embryos.

We used a dual-color MS2 and PP7 system to visualize endogenous *eve* transcription activity in wild-type and *Kr* heterozygous (*Kr^1^/+*) *Drosophila* embryos (Fig. 1A, Fig. S1A,B, Movie 1) (Garcia et al., 2013; Hocine et al., 2013; Lim et al., 2018a). The *eve-MS2* fly line contains a 24x MS2 sequence inserted at the 3′UTR of endogenous *eve* (Lim et al., 2018a). Upon transcription, the MS2 sequence forms a series of stem-loops and is bound by maternally loaded MCP-GFP molecules (Garcia et al., 2013). These clusters of MCP-GFP molecules can be observed as bright puncta in active transcription loci, indicating the activity of nascent transcripts (Fig. 1A). To distinguish *Kr* heterozygotes from wild types, we used the *iab5*>*PP7* reporter construct, where the *iab5* enhancer drives expression of the *PP7-yellow* reporter gene. *PP7* works in a similar way to *MS2*. Nascent transcripts of the *PP7-yellow* reporter can be visualized upon binding of maternally loaded PCP-mCherry proteins to PP7 stem loops (Hocine et al., 2013). Using this dual-color system, heterozygotes and wild types can be distinguished with the absence and presence of the *iab5>PP7* reporter gene expression, respectively (see Materials and Methods for details) (Fig. 1A). We confirmed that decreasing the *Kr* dosage by half has no significant impact on the AP and dorsal-ventral (DV) size of embryos (Fig. S1C-F). All seven *eve* stripes are formed by late NC14 for both wild-type and *Kr* heterozygous embryos (Fig. 1A,B). However, both spatial and temporal dynamics of the stripe formation are affected by the *Kr* dosage reduction in heterozygotes (Fig. 1B). We observed a wider *eve* stripe 2 around mid-NC14, delayed stripe 4 activation and a lower stripes 3 and 4 transcriptional activity in *Kr* heterozygous embryos compared with wild types (Fig. 1B, Fig. S2A,B). The mRNA production level is also decreased by the *Kr* dosage reduction (Fig. 2A, Fig. S2C). Moreover, we found that the *eve* stripe 3-5 domain is narrower in *Kr* heterozygotes than in wild types throughout the second half of NC14 (Fig. 2B), agreeing with previous studies (Frasch and Levine, 1987; Surkova et al., 2013).

In addition to overall changes in the spatiotemporal pattern, we observed a greater variability of *eve* expression in *Kr* heterozygous embryos. More than half of the embryos show pronounced changes in *eve* stripes 2-5 (Fig. 1B). However, a few heterozygous embryos exhibit minor differences, showing a more wild-type-like pattern of *eve* (Fig. S2D). We focused on the transcriptional activity of stripes 2-5, which overlap with the Kr expression domain, and quantified the degree of variability. A higher coefficient of variation (CV) was measured in *Kr* heterozygotes than in wild types, especially within the stripe 2 and 5 regions, and at the inter-stripe regions between stripes 2 and 3, and 3 and 4 (Fig. 2C,D). Indeed, *Kr* heterozygous and null larvae exhibit variable levels of defects in the number of body segments (Wieschaus et al., 1984), indicating the instability of the body patterning regulation.

### Decreased *Kr* directly affects the formation of *eve* stripes 2 and 5

**Fig. 3.**
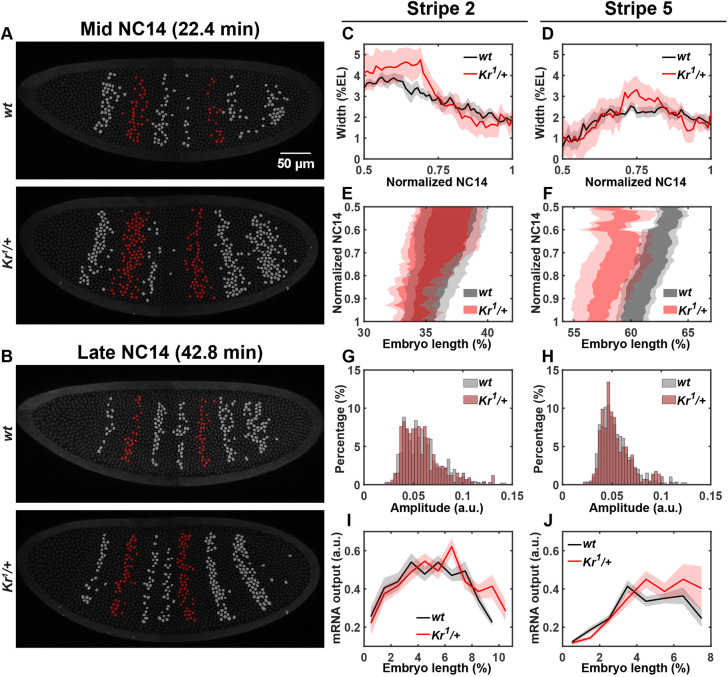
**Halving *Kr* dosage directly changes the boundary positions of *eve* stripes 2 and 5.** (A,B) False-colored wild-type and *Kr* heterozygous embryos. Transcriptionally active nuclei within the *eve* stripe 2 and 5 domains are in red; other active nuclei are in gray. (C,D) Average width of stripes 2 (C) and 5 (D) over time. (E,F) Average positions of stripes 2 (E) and 5 (F). Data are mean±s.e.m. in C-F for wild-type (*n*=7) and *Kr* heterozygous (*n*=5) embryos. (G,H) Distribution of the average transcriptional amplitude of individual nuclei in stripes 2 (G) and 5 (H). (I,J) Average mRNA output at mid-NC14 along the AP axis within stripe 2 (I) and stripe 5 (J) domains. 452 (stripe 2 *wt*), 328 (stripe 2 *Kr^1^/+*), 394 (stripe 5 *wt*) and 283 (stripe 5 *Kr^1^/+*) nuclei from seven wild-type and five *Kr* heterozygous embryos were analyzed for G-J.

Transcription of the seven *eve* stripes is regulated by five stripe-specific enhancers, and Kr directly binds to the stripe 2 and 5 enhancers (Fig. 1A) (Fujioka et al., 1999; Harding et al., 1989; Small et al., 1992; Small et al., 1996). As a result, reduced *Kr* dosage affects the spatial pattern of these two stripes. In mid-NC14, stripe 2 shows posterior expansion, such that the width of stripe 2 is ∼1.6 nuclei wider in *Kr* heterozygotes (Fig. 1B, cyan arrow). However, this expansion is only transient, as the stripe width becomes comparable between wild types and heterozygotes in late NC14 (Fig. 3A-C). We hypothesize that the reduced Kr concentration induces stripe 2 to expand in mid-NC14. In wild-type embryos, the *Kr* domain shifts anteriorly and the Kr protein level increases over NC14 (Crombach et al., 2012; El-Sherif and Levine, 2016; Surkova et al., 2008). If *Kr* heterozygous embryos follow the same trend of Kr expression dynamics as the wild types, the Kr expression domain would shift anteriorly and the level would go up in late NC14, although the level will still be lower than the wild-type Kr level at the same time point. This increase in Kr level may be sufficient to refine the expanded *eve* stripe 2 domain in *Kr* heterozygotes to match the wild-type width in late NC14 (Fig. 3B,C). Similarly, the *eve* stripe 5 domain is slightly wider in *Kr* heterozygous embryos, despite greater embryo-to-embryo variability (Fig. 3A,B,D). Due to the transient domain expansion (stripe 2) and high variability in width (stripe 5), the total number of transcriptionally active nuclei in stripe 2 and 5 domains remains comparable between wild types and heterozygotes (Fig. S3A-D). Reduced Kr concentration also shifted the position of stripe 5. Although the stripe 2 domain does not change significantly, stripe 5 is located approximately three nuclei closer to the anterior tip in *Kr* heterozygotes than in wild types (Fig. 3E,F).

Despite changes in boundary positions, the amount of mRNA produced by individual nuclei, their transcriptional amplitude and the duration of active transcription in these two stripes are similar between the wild-type and heterozygous embryos (Figs 2A and 3G,H, Fig. S3E-H). In both wild types and *Kr* heterozygotes, Kr represses *eve* in a dose-dependent manner. When we measure the mRNA production level of the stripe 2 and 5 domains along the AP axis during mid-NC14, we observe a gradual decrease in the posterior and anterior side of stripes 2 and 5, respectively (Fig. 3I,J). This indicates that the Kr expression gradients near the posterior and anterior border of stripes 2 and 5 repress *eve* as a function of its relative concentration. Our findings agree with a previous study demonstrating that the transcriptional activity of *eve* is lower in the nucleus with an intermediate Kr level, compared with the nucleus with a low Kr level (Bothma et al., 2018). In summary, our results indicate that stripe 2 and 5 enhancers are sensitive to the decrease in Kr concentration, but the impact of Kr is limited to shifts in boundary positions, with little effect on the mRNA production of individual nuclei located in both stripes.

### Decreased *Kr* indirectly hampers *eve* transcription in stripe 3 and 4 domains

**Fig. 4.**
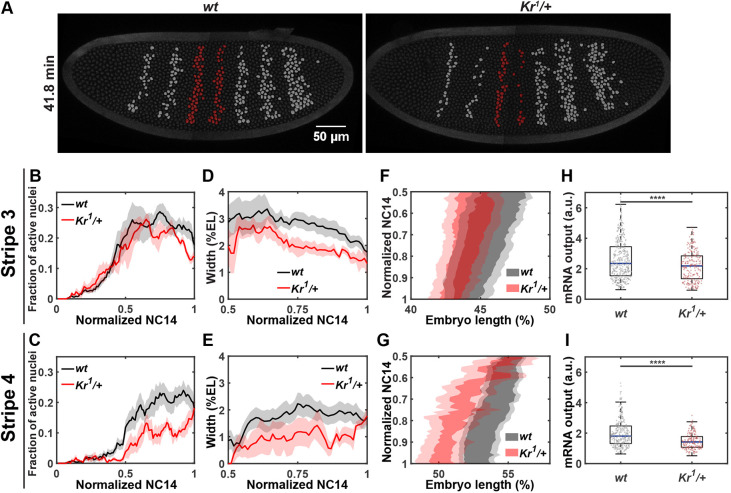
**Decreased *Kr* dosage affects the boundary positions and transcriptional activity of *eve* stripes 3 and 4.** (A) False-colored wild-type and *Kr* heterozygous embryos. Transcriptionally active nuclei within the *eve* stripe 3 and 4 domains are in red; other active nuclei are in gray. (B,C) Average activation kinetics of stripe 3 (B) and stripe 4 (C). (D,E) Average width of stripe 3 (D) and stripe 4 (E). (F,G) Average position of stripe 3 (F) and stripe 4 (G). Data are mean±s.e.m. (H,I) Cumulative mRNA output of individual nuclei within stripe 3 (H) and stripe 4 (I). The box represents the lower (25%) and upper (75%) quantile, and the blue bar denotes the median (50%). The whiskers extend from the zero to the 100th percentile of the distribution. Each point represents data from an individual nucleus. ****P<0.0001 (Student's *t*-test). 416 (stripe 3 *wt*), 227 (stripe 3 *Kr^1^/+*), 334 (stripe 4 *wt*) and 181 (stripe 4 *Kr^1^/+*) nuclei from seven wild-type and five *Kr* heterozygous embryos are analyzed.

We observed the most significant changes in stripes 3 and 4 (Figs 1B and 4A). Stripe 4 formation is delayed by ∼6 min, and this stripe comprises many fewer transcriptionally active nuclei (Fig. 1B - yellow star, Fig. 4C, Fig. S4B). Stripe 3 has a similar timing of activation in both wild types and heterozygotes, but fewer nuclei are activated in the domain of the heterozygotes (Fig. 4A,B, Fig. S4A). As a result, the widths of both stripes 3 and 4 are ∼0.9 nuclei narrower in the heterozygotes (Fig. 4D,E). We note that the nuclei located in the posterior region of stripe 3 are not activated in heterozygotes, thus affecting the posterior boundary of stripe 3 (Fig. 4F). For stripe 4, the entire expression domain shifts anteriorly in heterozygotes compared with wild types (Fig. 4G).

In addition to changes in spatial dynamics, fewer mRNAs are accumulated in stripe 3 and 4 regions in heterozygotes than in wild types (Fig. 2A, Fig. 4H,I). Further investigation suggests that this decrease is a result of lower transcriptional amplitude for stripe 3, and both shortened active transcriptional duration and lower transcriptional amplitude for stripe 4 (Fig. S4C-F). Moreover, we observed sporadic transcriptional activity of stripe 4 nuclei in *Kr* heterozygotes (Movie 2). This sporadic expression not only contributes to the most disrupted expression in stripe 4 compared with other affected stripes (Fig. 2A, Fig. S2C), but also causes the high variability in the spatial pattern of stripe 4 (Figs 4G and 2D).

The significant effects of *Kr* dosage modulation in *eve* expression are interesting because Kr does not directly regulate stripe 3 and 4 expression. ChIP-ChIP and ChIP-seq analyses show strong Kr binding at the *eve* stripe 2 and 5 enhancers, indicating a direct impact of Kr on *eve* stripes 2 and 5. However, there is no Kr binding at the 4+6 enhancer and very low Kr binding at the *eve* 3+7 enhancer (Castro-Mondragon et al., 2022; Li et al., 2008; Paris et al., 2013). Additionally, stripe 3 expression can be reconstructed without Kr *in silico* (Ilsley et al., 2013), suggesting the regulation of stripe 3 is independent of Kr. There exists a possibility that Kr transiently or weakly binds to the 4+6 and 3+7 enhancers below the ChIP-seq detection threshold level. However, direct Kr binding to enhancers would result in an expansion of stripe 3 and 4 expression domains upon decreasing Kr concentration because Kr acts as a repressor (Sauer and Jäckle, 1991). Instead, we observed a decrease in stripe 3 and 4 expression (Fig. 4); hence, we believe that *eve* stripes 3 and 4 are not directly regulated by Kr. ChIP-seq and misexpression analyses provided evidence of gap genes Hb and Kni directly repressing stripes 3 and 4 (Clyde et al., 2003; Paris et al., 2013). As mutual repression exists among Kr, Kni and Hb, the anterior shift of stripe 3 and 4 boundaries in *Kr* heterozygotes compared with wild types may be caused by an indirect effect of decreased *Kr* influencing its adjacent *kni* and *hb* domains (Capovilla et al., 1992; Clyde et al., 2003; Hülskamp et al., 1990; Sauer and Jäckle, 1991).

### Decreased *Kr* regulates *eve* stripes through adjacent gap genes

**Fig. 5.**
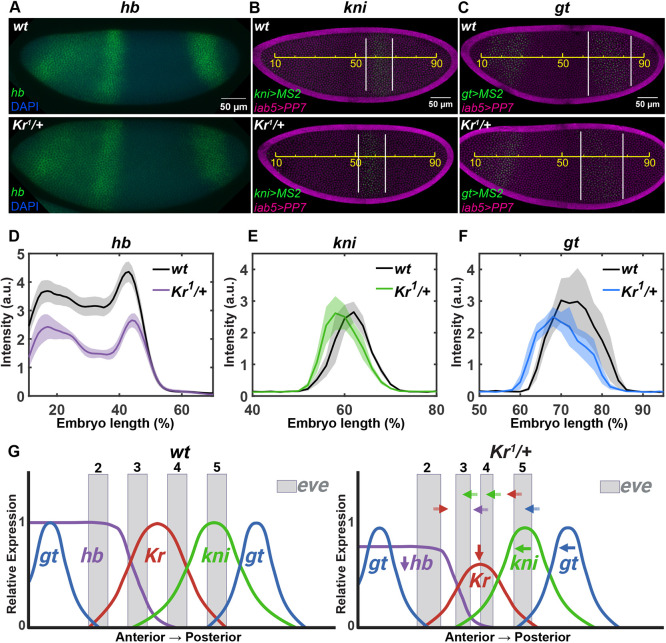
**Decreased *Kr* dosage affects adjacent gap genes.** (A) Wild-type and *Kr* heterozygous embryos stained for *hb* (green) and with DAPI (blue). (B,C) Wild-type and *Kr* heterozygous embryos expressing *kni>MS2* (green) and *iab5>PP7* (magenta) (B), and *gt>MS2* (green) and *iab5>PP7* (magenta) (C). Yellow line indicates the egg length; white lines indicate the *kni>MS2* or *gt>MS2* expression domain. (D-F) Spatial profiles of *hb* (D), *kni* (E) and *gt* (F) transcriptional activity. Data are mean±s.e.m. Ten, three and three wild-type, and seven, three and three *Kr* heterozygous embryos were used to visualize *hb, kni>MS2* and *gt>MS2*, respectively. There is one experimental replicate in D; results from another experimental replicate are shown in Fig. S5C. (G) A model illustrating how the halved *Kr* dosage affects gap genes and *eve* expressions. Decreased *Kr* shifts the posterior and anterior boundary of *eve* stripes 2 and 5, respectively (red arrows). Decreased *hb* in *Kr* heterozygotes induces an anterior shift of *eve* stripe 4 (purple arrow). An anterior shift of *kni* and *gt* induces the anterior shift of the posterior boundaries of stripes 3 and 4 (green arrows) and stripe 5 (blue arrow).

To further investigate this hypothesis, we used RNA fluorescence *in situ* hybridization (FISH) and live imaging to quantify the expression of *hb*, *kni* and *gt*: three gap genes that regulate the transcription of *eve* stripes 2-5. For all the gap genes analyzed, we assumed a linear correlation between mRNA and subsequent protein translation (Becker et al., 2013; Bothma et al., 2018). As both maternal and zygotic Hb regulate downstream genes, we performed FISH to visualize total *hb* mRNAs. In *Kr* heterozygotes, we observed a decrease in *hb* concentration in the anterior expression domain with no significant changes in the boundary position (Fig. 5A,D). It was shown that Kr has a dual regulatory role on *hb*: it works as an activator at the anterior *hb* expression domain where the Kr level is low, whereas it works as a repressor at the central region of an embryo where the Kr level is high (Holloway and Spirov, 2015; Sauer and Jäckle, 1991). In *Kr* homozygous mutant embryos, Hb expression was reduced, supporting the role of Kr as an activator for *hb* (Surkova et al., 2013). Our finding of a lower *hb* expression level in Kr heterozygotes agrees with these previous results. As the Hb repressor binds to the *eve* 4+6 enhancer, the decreased *hb* expression in *Kr* heterozygotes may cause the anterior shift of stripe 4 (Fig. 5G, purple) (Clyde et al., 2003).

We next examined changes in *kni* expression, as Kni directly defines the posterior boundaries of stripes 3 and 4 by binding to *eve* 3+7 and 4+6 enhancers (Clyde et al., 2003). We used the MS2-based live imaging of the *kni>MS2* transgenic line, where *kni* enhancer drives the expression of the *MS2-yellow* reporter gene (Syed et al., 2021). Spatial profiles of *kni* in *Kr* heterozygotes reveal a more anteriorly located domain compared with the wild types (Fig. 5B,E). This pushes the posterior boundaries of stripes 3 and 4 to the anterior side in *Kr* heterozygous embryos (Fig. 5G, green).

Lastly, we investigated the changes in *gt* expression dynamics upon *Kr* dosage modulation, using the *gt>MS2* transgenic line that contains the *gt* regulatory element from the *gt* promoter to 10 kb upstream of the promoter (Syed et al., 2021). As expected, the posterior *gt* stripe is expressed more anteriorly in the heterozygous embryos, because of the weaker repression by Kr (Fig. 5C,F) (Kraut and Levine, 1991). On the other hand, the anterior *gt* stripes remain unchanged (Fig. S5A,B) due to the lack of Kr-binding sites in the gt-23 enhancer that regulates anterior *gt* stripes (Hoermann et al., 2016; Ochoa-Espinosa et al., 2005). Gt directly binds to *eve* stripe 2 and 5 enhancers to regulate the anterior boundary of stripe 2 and the posterior boundary of stripe 5 (Fujioka et al., 1999; Small et al., 1991). Therefore, the anteriorly expressed *gt* posterior domain leads to the anterior shift of stripe 5 in *Kr* heterozygotes (Fig. 5G, blue). Meanwhile, the anterior boundary of stripe 2 remains comparable between heterozygotes and wild types because of the unaltered anterior *gt* domains. Although both stripes are directly regulated by Kr, this indirect effect through changes in *gt* expression contributes to a more significant effect on stripe 5 position compared with stripe 2*.*

## DISCUSSION

Although TFs play a pivotal role in gene regulation, changing the dosage of many TFs often leads to fertile and viable progeny. For example, female flies carrying one to four copies of the TF *dorsal* can produce normal offspring (Govind et al., 1993). In another study, increasing the level of a TF Bcd by ∼2.5-fold results in a twofold difference in the location of the cephalic furrow during embryogenesis, yet the embryos develop into normal flies (Liu et al., 2013). In mammalian systems, it has also been suggested that having multiple regulatory enhancers (and hence many binding sites of the same TF) confers phenotypic robustness such that loss of an enhancer does not affect viability (Frankel et al., 2010; Osterwalder et al., 2018). However, most studies have focused on the lethality of resulting phenotypes, and overlooked more mild changes in the expression of target genes or subtle changes in developmental phenotypes upon TF dosage changes.

Recent works suggest that mild perturbation of TF concentrations can result in significant phenotypic variations, without resulting in lethality (Lim et al., 2024; Naqvi et al., 2023). In human facial progenitor cells, some regulatory enhancers that are directly regulated by the TF SOX9 demonstrate sensitivity to *Sox9* dosage (Long et al., 2020; Naqvi et al., 2023). Although many genes show minimal changes upon *Sox9* dosage modulation, some key pro-chondrogenic genes, especially those that are associated with craniofacial development, exhibit heightened sensitivity to *Sox9* dosage. A 25% reduction in SOX9 level results in a shape variation of lower jaw development within the normal range, and a 50% reduction leads to more significant craniofacial malformations (Naqvi et al., 2023). Similarly, a slight increase in the binding affinity of a TF ETS binding site in the ZRA enhancer of *Sonic hedgehog* results in polydactyly, indicating the sensitivity to TF level (Lim et al., 2024). These results suggest that some sets of genes do exhibit a higher sensitivity to TF concentrations than others, leading to a significant impact on developmental phenotypes. Hence, it is important to quantitatively analyze how gene expression changes upon TF level modulation, and examine its relationship with phenotypic differences.

In this study, we used the *Drosophila* gap and pair-rule gene-mediated AP patterning event as a model system to examine the molecular impact of TF dosage modulation in target gene regulation. We have quantitatively measured the transcriptional activity of endogenous *eve* in wild-type and *Kr* heterozygous *Drosophila* embryos. In *Kr* heterozygotes, *eve* stripe 2 exhibits a transient posterior expansion around mid-NC14, and the stripe 5 expression domain is anteriorly shifted compared with wild types. Strikingly, although the stripe 3 and 4 enhancers do not contain Kr-binding sites (Castro-Mondragon et al., 2022; Li et al., 2008; Paris et al., 2013), we observed the most significant changes in these two stripes: both stripes have narrower expression domains and are located more anteriorly in heterozygotes compared with wild-type embryos. In addition, decreased Kr concentration hampers the transcriptional competency of both stripes, resulting in reduced mRNA production. We demonstrate that reducing Kr dosage affects stripe 3 and 4 expression indirectly, by changing the spatiotemporal dynamics of other gap genes regulated by Kr. These changes in *eve* expression correlate with the patterning phenotypes in thorax and abdomen segment formation in *Kr* heterozygous larvae (Wieschaus et al., 1984).

As the *eve* stripe 2 enhancer is one of the most well-characterized and thoroughly studied enhancers, many previous papers have examined the role of Kr repression on *eve* stripe 2. In *Kr* null embryos, the *eve* stripe 2 expression domain shifts posteriorly, with a larger expansion of the posterior border (Frasch and Levine, 1987; Surkova et al., 2013; Warrior and Levine, 1990). However, studies on reporter constructs show discrepancies in *eve* stripe 2 enhancer-driven expression. In *Kr* null or Kr-binding site mutation backgrounds, *eve* stripe 2 expression was expanded significantly (Small et al., 1991), only mildly (Stanojevic et al., 1991) or not at all (Small et al., 1992), depending on the size of the enhancers used. This emphasizes the need to analyze changes in gene expression in the endogenous context. By visualizing endogenous *eve* expression in *Kr* heterozygous embryos, we observed a transient expansion of the *eve* stripe 2 compared with wild types, consistent with previous results.

The impact of Kr dosage modulation can be manifested all the way to later development to exhibit body segmentation phenotypes. In most *Kr* homozygous mutant larvae, regions between the head and the sixth abdominal segment are deleted and substituted with a reversed sixth abdominal denticle band, missing the entire thorax and anterior abdominal segments (Wieschaus et al., 1984). A milder phenotype is observed in *Kr* heterozygotes. There is no polarity reversal, and all thorax and abdominal segments exist. However, *Kr* heterozygous larvae still show varying degrees of denticle band defects in mesothorax, metathorax and the second abdominal segment (Wieschaus et al., 1984). The differences in cuticle phenotypes are supported by the respective changes in *eve* and gap gene expression patterns in *Kr* null and heterozygous embryos. For example, the posterior *gt* expression domain is shifted more anteriorly in *Kr* null mutants compared with the shift observed in *Kr* heterozygous embryos (Surkova et al., 2019). In addition, Kni expression is significantly decreased in *Kr* null, while we observe no significant changes in *kni* expression level in *Kr* heterozygotes (Surkova et al., 2013) (Fig. 5E). These more pronounced changes in gap gene expression in *Kr* null embryos affect downstream *eve* expression patterns more drastically, leading to the fusion of *eve* stripes 2 and 3, and 4 to 6, and the subsequent loss of body segments observed in *Kr* null larvae (Frasch and Levine, 1987; Wieschaus et al., 1984). On the other hand, *eve* expression is affected only mildly in *Kr* heterozygotes, with the biggest impact on stripes 3 and 4 (Figs 3 and 4). Indeed, the denticle band defects observed in *Kr* heterozygotes correspond to the domains of *eve* stripes 3 and 4 (Lawrence et al., 1987; Martinez-Arias and Lawrence, 1985). Our analysis shows how *Kr* dosage confers sensitivity to the target gene *eve* in an indirect manner, through other gap genes, and eventually affects body segment phenotypes. Many *in silico* mathematical models exist to investigate the contribution of each TF on individual *eve* stripe expression and make predictions under various genetic backgrounds (Ilsley et al., 2013; Janssens et al., 2006; Kozlov et al., 2012). Future study can incorporate these heterozygous datasets and establish a more comprehensive mathematical framework of TF-mediated gene regulation.

Taken together, our study quantitatively analyzes how modulating the dosage of Kr, a key TF, affects the target gene *eve* expression through multifaceted direct and indirect interactions. Whereas the direct regulation of Kr alters the positions of *eve* stripe 2 and 5 boundaries, indirect regulation via changes in other gap gene dynamics leads to a more dramatic effect on both the transcription of *eve* stripes and body patterning (Fig. 5G). In many circumstances, the determination of cell fate requires a balanced input of several mutually regulating TFs (Govind et al., 1993; Jaeger et al., 2004; Li and Belmonte, 2017). This interplay of the regulatory network implies that a shift in cell fate often results from changes in multiple TFs, propagated by a change in a single TF. Our results suggest that both the spatial and temporal patterning of early *Drosophila* embryos are sensitive to fluctuations in TF concentration, and the phenotypic consequences result from more convoluted interactions among multiple proteins and their intricate dynamics.

## MATERIALS AND METHODS

### Fly strains

Existing *eve-MS2* (Lim et al., 2018b), *gt>MS2-yellow* (*gt>MS2*) (Syed et al., 2021), *kni>MS2-yellow* (*kni>MS2*) (Syed et al., 2021), *nos>MCP-GFP, mCherry-PCP, His2Av-eBFP* (*MCP, PCP, His2Av*) (Lim et al., 2018a), Sp/CyO; Dr/TM3 (Bloomington Stock Center, #59967), and *Kr^1^/SM6* (Bloomington Stock Center, #3494) fly lines were used in this study. The iab5>*snaPr-PP7-yellow* (*iab5>PP7*) reporter construct was generated by using the iab5 enhancer (Zhou et al., 1996), 100 bp core snail promoter and *PP7-yellow* reporter gene (Keller et al., 2020). The plasmid was targeted to the VK02 locus in the second chromosome using PhiC-mediated site-directed integration (Venken et al., 2006).

### Imaging *eve-MS2* in wild-type and *Kr* heterozygous embryos

*iab5>PP7* virgin females were mated with Sp/CyO; Dr/TM3 males. *MCP, PCP, His2Av* virgin females were mated with Sp/CyO; Dr/TM3 males in parallel. The resulting *iab5>PP7/CyO; +/Dr* and *+/Sp; MCP, PCP, His2Av/TM3* offspring generated from these two parallel crosses were then mated and homogenized to produce stable strain of *iab5>PP7* ; *MCP, PCP, His2Av*. *iab5*>*PP7*; *MCP, PCP, His2Av* virgin females were mated with *Kr^1^/SM6* males to generate wild-type and *Kr* heterozygous embryos. The resulting *Kr^1^/iab5>PP7 ; +/MCP, PCP, His2Av* virgin female progenies were selected to mate with *eve-MS2* males. Embryos laid from this cross that inherit the *Kr^1^* allele are heterozygous for *Kr*, whereas embryos that inherit the wild-type allele are wild-type embryos and can be recognized by the *iab5>PP7* reporter gene expression. Both wild-type and *Kr* heterozygous embryos carry one copy of *eve-MS2* for visualization of *eve* expression.

### Imaging gap genes in wild-type and *Kr* heterozygous embryos

*Kr^1^/iab5>PP7 ; +/MCP, PCP, His2Av* were generated using the same steps in the last section. The virgin females were collected and mated with *kni>MS2* or *gt>MS2* males. Embryos from this cross were used for imaging. Similarly, wild-type embryos were identified by the expression of *iab5>PP7*, while *Kr* heterozygous embryos have no *iab5>PP7* transcription. The embryos from crossing *Kr^1^/iab5>PP7; +/MCP, PCP, His2Av* with *gt>MS2* were also used in fluorescent *in situ* hybridization to detect *hb* and *Kr* mRNA.

### Live imaging

Embryos were collected 2 h after being laid. They were dechorionated with 50% bleach for 2.75 min and mounted in Halocarbon oil 27 (Sigma) between a semipermeable membrane (Sarstedt) and coverslip (18 mm×18 mm). All movies were imaged at room temperature (∼23°C) with a Zeiss LSM800 confocal laser scanning microscope and Plan-Apochromat 40×/1.3 numerical aperture (N.A.) oil-immersion objective. 408 nm, 488 nm and 561 nm lasers were used to visualize His2Av-eBFP2, MCP-GFP and mCherry-PCP, respectively. To capture the full embryo, two adjacent tiles with 50 pixel overlaps were taken, resulting in a final image size of 950×500 pixels. A stack of 15 images with 0.7 µm step size in *z* were captured at each time point with a time resolution of 61 s/frame. For *eve-MS2*, seven biological replicates were taken for the wild-type embryos and five were taken for the *Kr* heterozygous embryos. Three replicates were taken for the wild-type and *Kr* heterozygous embryos for *kni>MS2* and *gt>MS2*. The same laser setting was used between wild-type and *Kr* heterozygous embryos.

### Fluorescence *in situ* hybridization

Embryos were collected 3 h after being laid and were fixated with formaldehyde. Fluorescence *in situ* hybridization was performed using previously published protocols (Lim et al., 2015). *Kr*-DIG, *hb*-FITC, *eve*-Biotin, *yellow-*Biotin RNA probes and Alexa Fluors were used to visualize *Kr*, *hb*, *eve* and *yellow* expression. Sheep anti-DIG (11093274910, Roche), rabbit anti-FITC (A889, Invitrogen) and mouse anti-Biotin (200-002-211, Jackson Immuno Research) were used as primary antibodies, Alexa Fluor donkey anti-rabbit 488 (A32790, Invitrogen), donkey anti-sheep 555 (A21436, Invitrogen) and donkey anti-mouse 647 (A32787, Invitrogen) were used as secondary antibodies, and DAPI (D1306, Invitrogen) was used to stain nuclei. *yellow* probe was used to visualize *iab5>PP7-yellow*. Embryos were imaged with a Zeiss LSM800 confocal laser scanning microscope and Plan-Apochromat 20×/0.8 N.A. objective. Single image size was set to 1024×1024 pixels. Only the laterally oriented embryos in mid-NC14 were analyzed to minimize the noise due to orientation and developmental stage variations. Twenty-four biological replicates were taken for the wild-type embryos; 17 were taken for the *Kr* heterozygous embryos from two experimental replicates.

### Image analysis

Image processing and data analysis were performed in FIJI (ImageJ) and MATLAB (R2021b), using custom image analysis code (Keller et al., 2020). Images with maximum projections of *z*-stacks were used for analysis.

### Live imaging

Nuclei segmentation, nuclei tracking and signal measurement were adapted from Keller et al. (2020). MS2 signal within each nucleus was measured as the average of the two pixels with the highest fluorescent intensity from a max-projected image. To minimize the variation in DV length, we extracted transcriptional activity from nuclei within a fixed DV region of 282 µm, spanning ∼17 nuclei in the middle of all embryos. To normalize the AP length differences, each gene expression domain was measured as % egg length (EL). EL was measured as the distance between the anterior and posterior tips of each embryo. Each embryo was divided into 50 EL bins (2%) along the AP axis. Gene expression profile along the AP axis was quantified as the average of the signal intensity of all nuclei within each bin at a given frame. Nuclei expressing active transcription in the last frame before gastrulation were used to define mature stripes. Each stripe domain is defined as the region between the anterior border of one mature stripe and the anterior border of the next mature stripe (e.g, the region between the anterior border of stripe 1 and the anterior border of stripe 2 is defined as the ‘stripe 1 domain’). The stripe 7 domain is defined as the region between the anterior border of stripe 7 and the posterior end of the embryo.

As each embryo has a slightly different duration of NC14, the time from the end of the 13th mitosis to the beginning of gastrulation was normalized to 100 time points on a 0-1 scale, except for representative embryo images. Time after the onset of NC14 is labeled for snapshots of representative embryos (Figs 1A,B, 3A,B and 4A). mRNA production of individual nuclei was approximated by taking the integration of the area under the MS2 fluorescent intensity trajectory over time. mRNA production along the AP axis was measured as the average mRNA production of all nuclei within each bin. To measure the mRNA output along the AP position within *eve* stripe 2 and 5 regions (Fig. 3I,J), the stripe domains from each embryo are aligned by their anteriormost nuclei. The average mRNA output corresponds to the time interval representing 61-70 points out of the normalized 100 time points during NC14, for each 1% EL bin within the stripe domains. 1% EL bins with fewer than 10 nuclei were excluded from measurement.

At each time point, active nuclei were defined with an MS2 signal above a threshold value. Average transcriptional amplitudes were calculated as the mRNA output divided by the duration of actively transcribing time points. To track the boundary positions and width of each *eve* stripe over time, embryos were divided into five sections along the DV axis. AP axis positions of the most anteriorly and posteriorly located transcriptionally active nuclei were marked for each section. The average position of the five sections was used as a boundary position for each stripe. The stripe width was measured as the average distance between the anterior and posterior boundaries of each stripe. The posterior expansion of stripe 2 is measured by the largest difference in stripe width around mid-NC14 in heterozygous embryos and wild types. Stripe positions and stripe widths during 80-90 time points based on the normalized NC14 (out of 100) are used to quantify differences in the average position of stripe 5, the average width of stripe 3, and the average width of stripe 4 in heterozygotes and wild types. For Fig. 5B,C, the boundaries for *kni* and *gt* posterior domains were set at the positions where the binned MS2 signal of *kni* or *gt* went above a threshold value.

### Fluorescent *in situ* hybridization

The middle 40 pixels along the DV axis of each embryo image were used to measure *hb* and *Kr* RNA signal intensity. Embryos were divided into 1% EL bins along the AP axis, and the signal intensities of all pixels within each bin were averaged. To minimize the effect of background noise, *hb* and *Kr* signals were subtracted by the lowest value from 10-90% EL.

## Supplementary Material

10.1242/develop.202132_sup1 Supplementary information
