# Supplementary material for: Multifaceted effects on even-skipped transcriptional dynamics upon Krüppel dosage changes
Source: Development. 2024 Mar 4;151(5):dev202132. doi: 10.1242/dev.202132 (PMC10948998; doi:10.1242/dev.202132)
Supplement: Supplementary information [file develop-151-202132-s1.pdf]

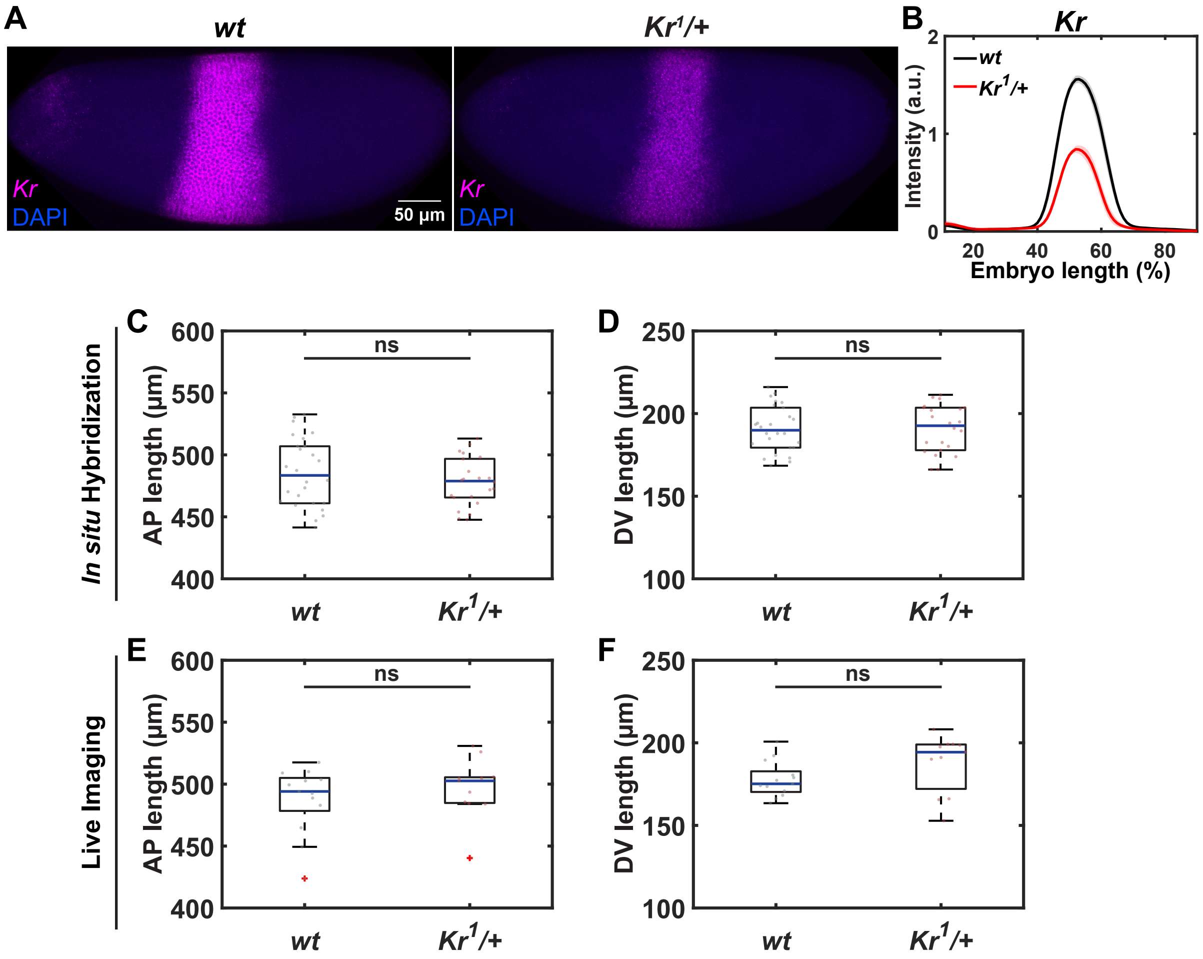

Fig. S1. Decreased *Kr* dosage has no effect on embryo size. (A) FISH of mid-NC14 wild-type and *Kr* heterozygous embryo stained with *Kr* (magenta) and DAPI (blue). (B) Average spatial profile of *Kr* signal intensity. Shaded error bars show the mean ± s.e.m. of 10 wild-type and 7 *Kr* heterozygous embryos from FISH. (C-D) The AP (C) and DV (D) lengths of embryos used in FISH. 26 wild-type and 22 *Kr* heterozygous embryos were analyzed. (E-F) The AP (E) and DV (F) lengths of embryos used in live-imaging. 13 wild-type and 11 *Kr* heterozygous embryos were analyzed. Scatters represent individual embryos. ns, not significant from the Student's t-test.

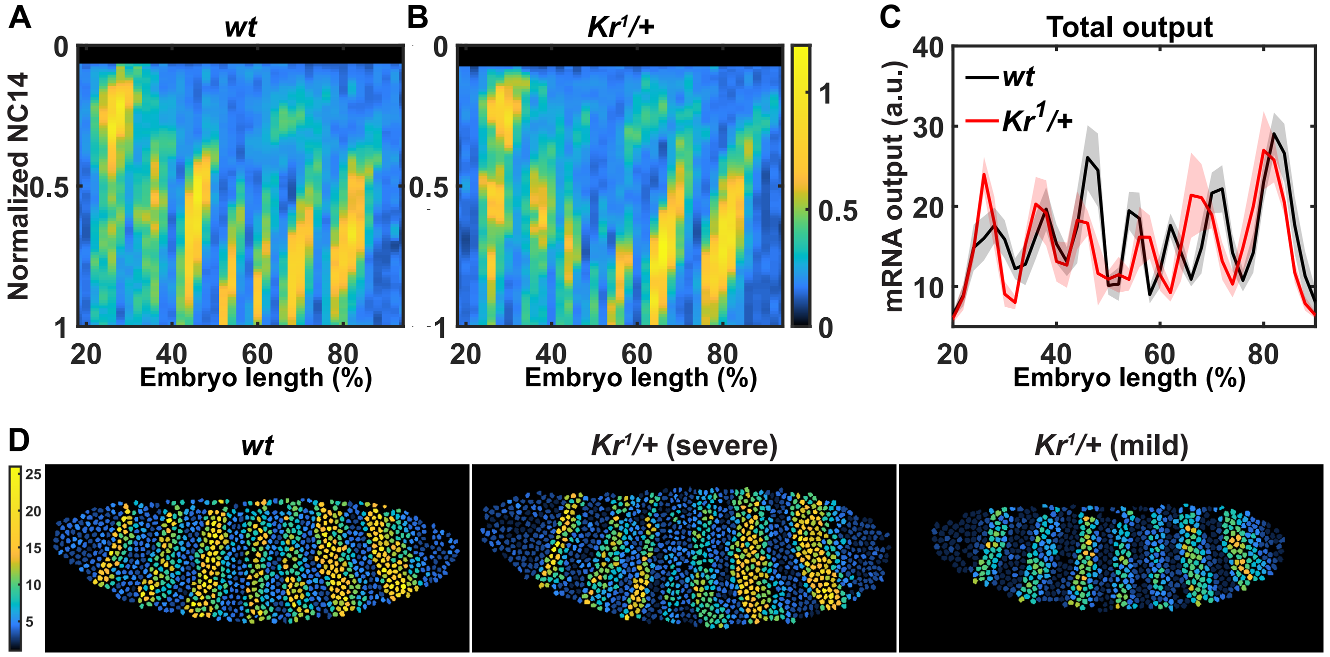

Fig. S2. Decreased *Kr* dosage changes *eve* dynamics. (A-B) Heatmaps showing the average spatial profile of *eve-MS2* signal intensity in a wild-type (A) and a *Kr* heterozygous embryo (B). (C) Average spatial profile of *eve* mRNA production in the second half of NC14. Shaded error bars show the mean ± s.e.m. of 7 wild-type and 5 *Kr* heterozygous embryos from live imaging. (D) Heatmaps showing cumulative mRNA production of individual nuclei in a wild-type (left) and *Kr* heterozygous embryos (middle and right). The embryo in the middle panel represents a *Kr* heterozygous embryo undergoing more pronounced changes in *eve* patterning. The embryo in the right panel represents a *Kr* heterozygous embryo with a more wild-type-like pattern of *eve*.

Development • Supplementary information

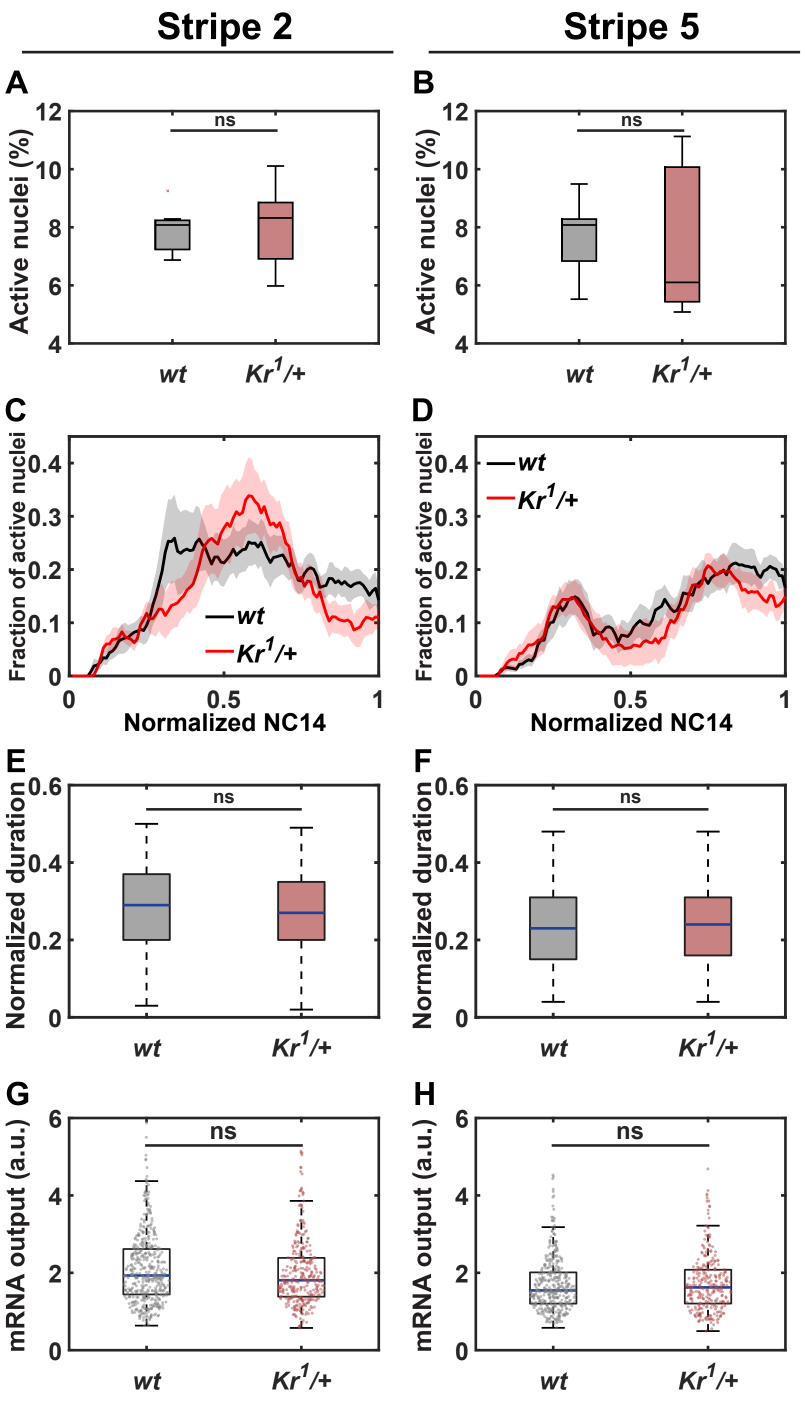

Development • Supplementary information

Fig. S3. Decreased *Kr* dosage has limited effect on *eve* stripes 2 and 5 mRNA production. (A-B) Boxplots showing the percentage of transcriptionally active nuclei in stripe 2 (A) and stripe 5 (B), out of the total number of nuclei being analyzed in each embryo. (C-D) Average activation kinetics of stripe 2 (C) and stripe 5 (D). Shaded error bars represent mean ± s.e.m.. (E-F) Boxplots showing the transcriptionally active duration of individual nuclei in stripe 2 (E) and stripe 5 (F). (G-H) Cumulative mRNA output of individual nuclei within stripe 2 (G) and 5 (H). Scatters represent individual nuclei. ns, not significant from the Student's t-test. The numbers of nuclei analyzed are 452 (stripe 2 *wt*), 328 (stripe 2 Kr^1/+), 394 (stripe 5 *wt*), and 283 (stripe 5 Kr^1/+). 7 wild-type and 5 *Kr* heterozygous embryos were analyzed.

Development • Supplementary information

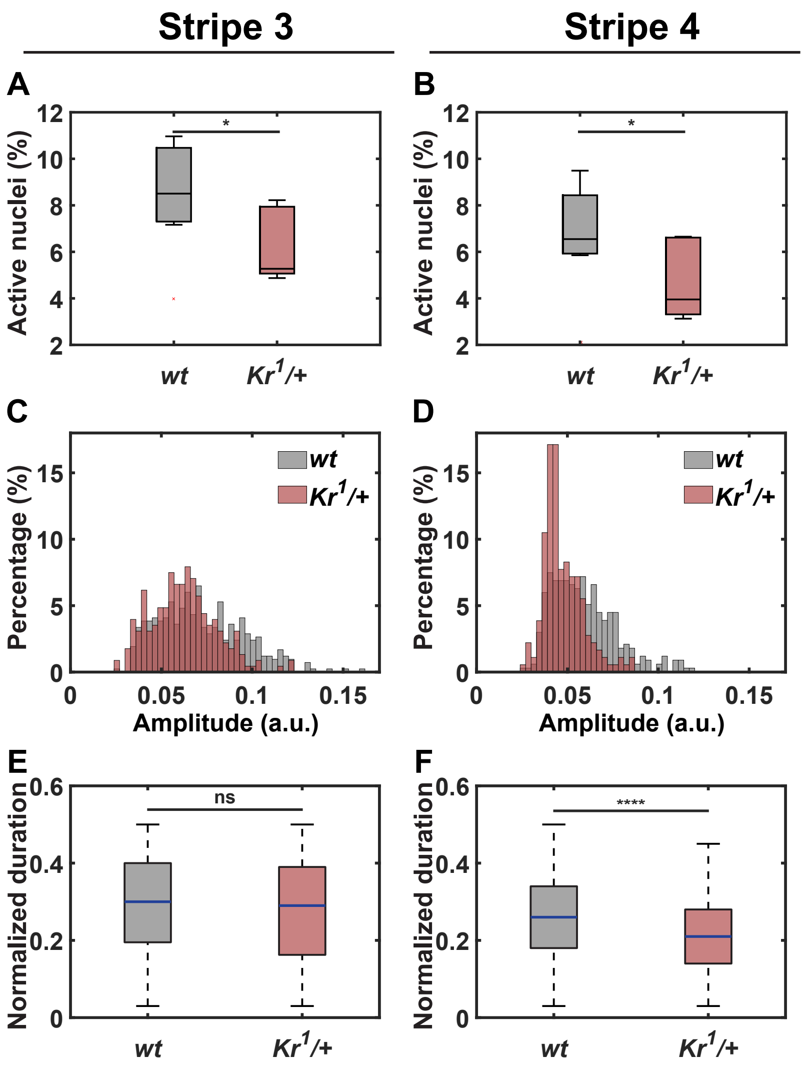

Fig. S4. Decreased *Kr* dosage affects *eve* stripes 3 and 4 expression. (A-B) Boxplots showing the percentage of transcriptionally active nuclei in stripe 3 (A) and stripe 4 (B), out of the total number of nuclei being analyzed in each embryo. (C-D) Histograms showing the distribution of the average transcriptional amplitude of individual nuclei in stripe 3 (C) and stripe 4 (D). (E-F) Boxplots showing the transcriptionally active duration of individual nuclei in stripe 3 (E) and stripe 4 (F). *$P<0.05$ and ****$P<0.0001$ from the Student's t-test. The numbers of nuclei analyzed are 416 (stripe 3 *wt*), 227 (stripe 3 *Kr*^{1}/+), 334 (stripe 4 *wt*), and 181 (stripe 4 *Kr*^{1}/+). 7 wild-type and 5 *Kr* heterozygous embryos were analyzed.

Development • Supplementary information

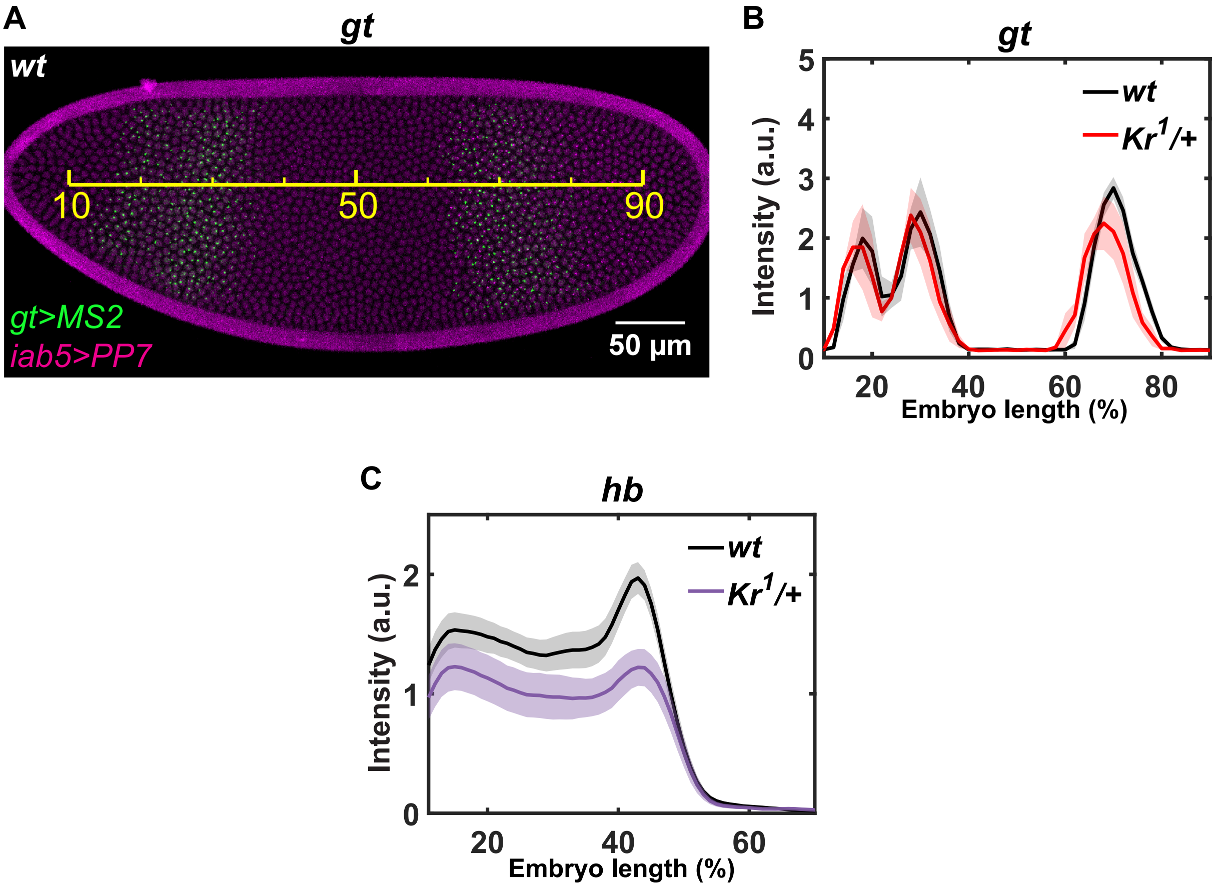

Fig. S5. Decreased *Kr* dosage affects the posterior *gt* domain. (A) Snapshot of a wild-type embryo expressing *gt>MS2* (green) and *iab5>PP7* (magenta). Yellow scale bar represents the estimated EL across the AP axis. (B) Average spatial profile of *gt* signal intensity. Shaded error bars show the mean ± s.e.m.. 3 wild-type and 3 *Kr* heterozygous embryos were analyzed. (C) Average spatial profile of *hb* signal intensity. This is an additional experimental replicate of the plot shown in Fig. 5D. Shaded error bars show the mean ± s.e.m.. 14 wild-type and 10 *Kr* heterozygous embryos were analyzed.

Development • Supplementary information

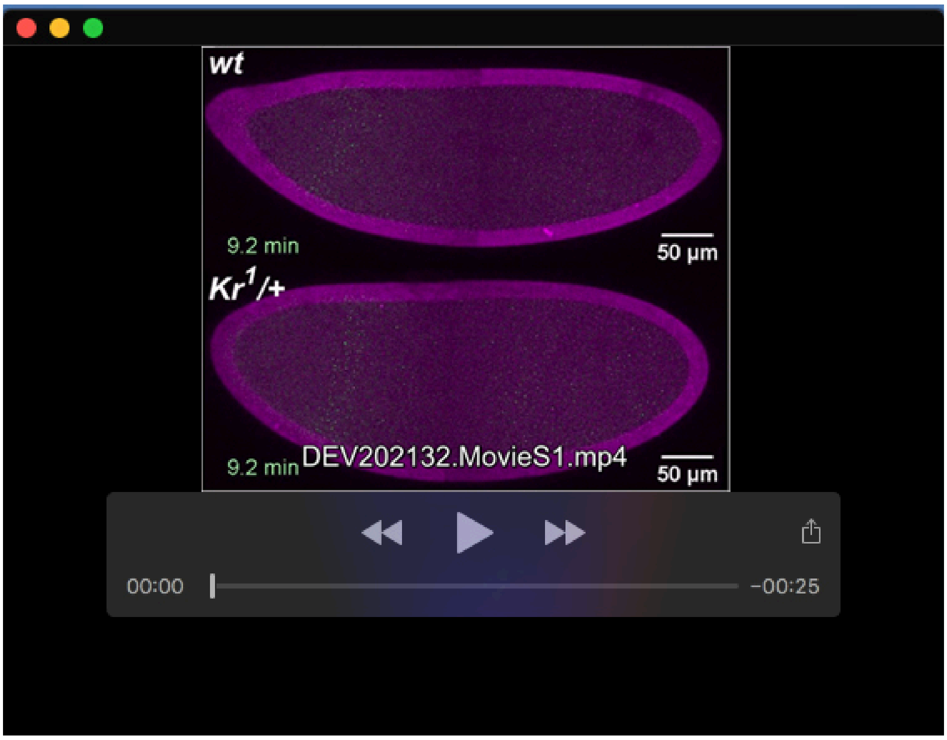

Movie 1. Live imaging of *eve-MS2* in wild-type (top) and *Kr* heterozygous (bottom) embryos during NC14. *eve-MS2* signal is shown in green. *iab5>PP7* signal is shown in magenta. Embryos are oriented left-anterior, right-posterior.

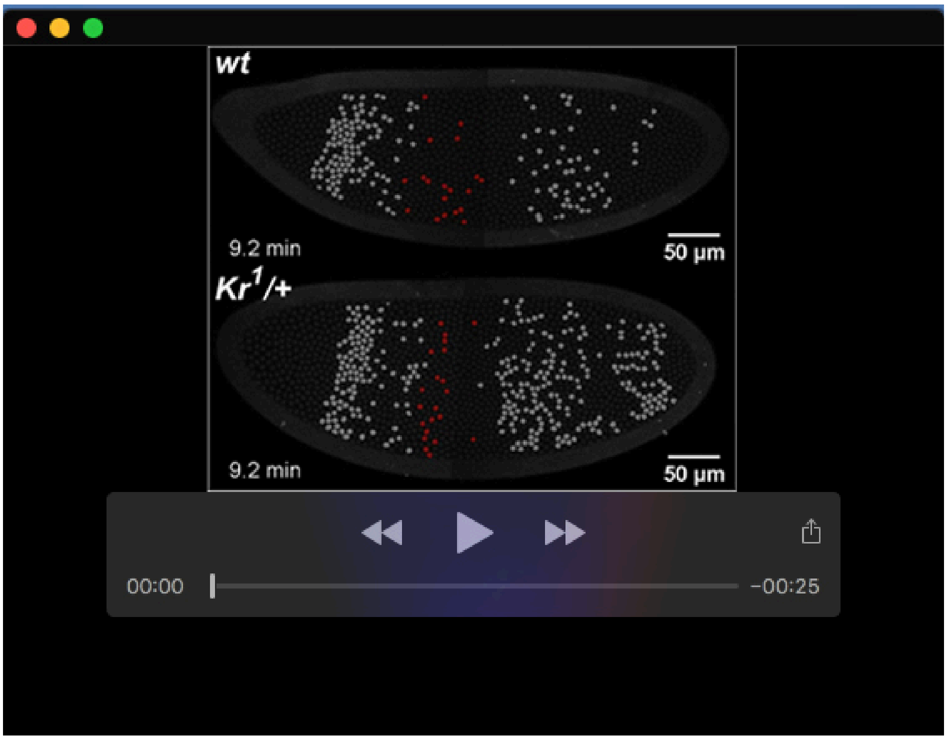

Movie 2. Live imaging of wild-type (top) and *Kr* heterozygous (bottom) embryos during NC14. Transcriptionally active nuclei within the *eve* stripe 3 and 4 regions are false-colored in red, and other active nuclei are false-colored in gray. Embryos are oriented left-anterior, right-posterior.
